# Measurement of oxygen consumption rates of human renal proximal tubule cells in an array of organ-on-chip devices to monitor drug-induced metabolic shifts

**DOI:** 10.1038/s41378-022-00442-7

**Published:** 2022-09-29

**Authors:** Samuel H. Kann, Erin M. Shaughnessey, Jonathan R. Coppeta, Hesham Azizgolshani, Brett C. Isenberg, Else M. Vedula, Xin Zhang, Joseph L. Charest

**Affiliations:** 1 Draper Scholar, 555 Technology Square, Cambridge, MA 02139 USA; 2 Department of Mechanical Engineering, Boston University, 110 Cummington Mall, Boston, MA 02215 USA; 3 Department of Biomedical Engineering, Tufts University, 4 Colby Street, Medford, MA 02155 USA; 4 Draper, 555 Technology Square, Cambridge, MA 02139 USA; 5 Present Address: Biogen, 225 Binney Street, Cambridge, MA 02142 USA

**Keywords:** Engineering, Chemistry

## Abstract

Measurement of cell metabolism in moderate-throughput to high-throughput organ-on-chip (OOC) systems would expand the range of data collected for studying drug effects or disease in physiologically relevant tissue models. However, current measurement approaches rely on fluorescent imaging or colorimetric assays that are focused on endpoints, require labels or added substrates, and lack real-time data. Here, we integrated optical-based oxygen sensors in a high-throughput OOC platform and developed an approach for monitoring cell metabolic activity in an array of membrane bilayer devices. Each membrane bilayer device supported a culture of human renal proximal tubule epithelial cells on a porous membrane suspended between two microchannels and exposed to controlled, unidirectional perfusion and physiologically relevant shear stress for several days. For the first time, we measured changes in oxygen in a membrane bilayer format and used a finite element analysis model to estimate cell oxygen consumption rates (OCRs), allowing comparison with OCRs from other cell culture systems. Finally, we demonstrated label-free detection of metabolic shifts in human renal proximal tubule cells following exposure to FCCP, a drug known for increasing cell oxygen consumption, as well as oligomycin and antimycin A, drugs known for decreasing cell oxygen consumption. The capability to measure cell OCRs and detect metabolic shifts in an array of membrane bilayer devices contained within an industry standard microtiter plate format will be valuable for analyzing flow-responsive and physiologically complex tissues during drug development and disease research.

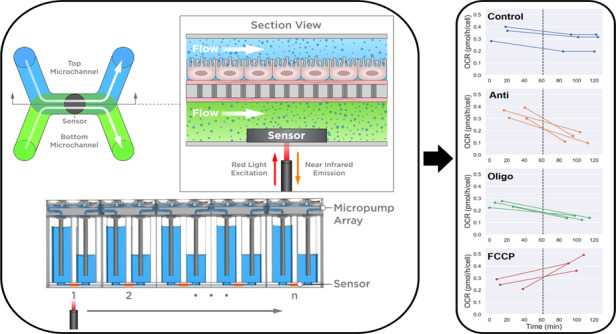

## Introduction

Drug development remains expensive and inefficient largely due to a lack of in vitro cell culture models and animal models that can accurately predict a drug’s effect in humans during clinical trials^1^. Microfluidic organ-on-chip (OOC) technology, a promising alternative to traditional in vitro models, allows for culturing of human tissue with precise, continuous, and long-term control of physical and chemical cues relevant to the organ under investigation^2^. Different OOC systems, including kidney-on-chip^3^, lung-on-chip^4,5^, and heart-on-chip^6,7^, have shown that the inclusion of relevant physiological cues, such as fluid flow and mechanical stress, can alter tissue morphology^8^, function^9^, and response to drug exposures^10^, resulting in in vitro tissue with improved human relevance compared to cells cultured in traditional systems, such as static 2D well plates.

Unfortunately, data collection from OOC systems still relies heavily on fluorescent imaging or colorimetric assays that are often focused on end points, require labels or added substrates, and generally lack kinetic data regarding cell culture conditions or cellular activity^11^. Integration of sensors within OOC systems has enabled label-free and real-time collection of key cell culture readouts, including oxygen levels, pH, and transepithelial/endothelial electrical resistance^12^. The measurement of oxygen is particularly critical in OOC systems because oxygen availability heavily influences cell function^13^, and the cell oxygen consumption rate (OCR) serves as a dynamic indicator of metabolic activity and mitochondrial function^14^. For the kidney, renal proximal tubule epithelial cells rely primarily on oxygen consumption via the mitochondria to generate sufficient energy to maintain physiological functions, including fluid/electrolyte balance and drug clearance^15^. Furthermore, studies have linked drug-induced nephrotoxicity and common renal diseases, including acute kidney injury and diabetic nephropathy, to mitochondrial dysfunction occurring within renal proximal tubule epithelial cells^16,17^. Because mitochondria are a key target in drug development, there is a need for oxygen sensor-integrated OOC systems that allow online and label-free measurement of the cell OCR to detect shifts in mitochondrial function resulting from drug treatments.

Electrochemical and optical-based sensing are two main approaches for label-free measurement of cell oxygen consumption in cell culture systems. While electrochemical-based sensing has been implemented in several static and microfluidic cell culture systems for oxygen consumption measurements, such systems are low throughput^18–20^. The limited scalability of electrochemical-based OOC systems is often attributed to the large footprint of the electrical hardware, complex fabrication processes, and the need for a reference electrode^21^. Optical-based sensors generally provide a low footprint, fast sensor response times, and straightforward integration with both static well plates and microfluidic devices^22,23^. Several optical-based systems, such as the commercial Agilent Seahorse XFeAnalyzer, offer high-throughput cell oxygen consumption measurements in traditional well plates^24,25^. However, they are not compatible with OOC systems and do not support perfusion during measurements. For these reasons, several groups have incorporated optical-based sensors in OOC systems to measure oxygen and cell oxygen consumption^26–28^. Unfortunately, existing systems contain only one or a few cell culture chambers situated in parallel, lack a microtiter plate format well suited for typical biological workflows, and have large footprints. Due to the above issues, the broad adoption of OOC systems in life science research and drug development has been limited. In recent years, several groups have arrayed up to 96 OOC devices in microtiter plate formats^29,30^; however, such systems have not yet demonstrated label-free measurement of the cell OCR. Recently, our group integrated optical-based oxygen sensor units within a high-throughput OOC platform, in which we measured oxygen consumption on the timescale of hours with low temporal resolution. The low temporal resolution of these measurements did not allow the quantification of cell OCRs or metabolic shifts^29^. Despite recent progress in the integration of oxygen sensors in scalable OOC systems, approaches for the measurement of cell OCRs and drug-induced metabolic shifts in such systems have yet to be reported. Additionally, cell OCR has yet to be measured in OOC systems that utilize a membrane bilayer format, in which cells are cultured on a porous membrane suspended between two microchannels, a common OOC architecture used to study human tissue barriers^8,31^. More specifically, localization of cells between two microchannels offers unique advantages for drug studies, including independent fluidic access to both the apical and basolateral surfaces of the tissue layer^32^, independent control of flow rates^29^, and compatibility with barrier-specific functional assays^33,34^.

Here, we report an approach to measure cell OCRs and drug-induced metabolic shifts in an array of membrane bilayer devices contained within an oxygen sensor-integrated microfluidic culture plate (O-MCP) in a microtiter plate format and industry-standard footprint. Each O-MCP device contained human renal proximal tubule epithelial cells (hRPTECs) cultured on a porous membrane between two microchannels with an optical-based sensor unit located in the bottom microchannel. The cells were exposed to continuous unidirectional perfusion and shear stress of 0.07 Pa, mimicking the flow conditions of the kidney’s proximal tubule segment^35^. We established a technique to measure oxygen depletion, changes in oxygen at the sensor in the bottom microchannel, across multiple devices by repeatedly turning flow on and off and using a programmable stage to align an optical fiber beneath each device’s sensor unit. A finite element analysis-based model of oxygen transfer was established to estimate cell OCRs from the measured oxygen depletion curves, which allowed comparison with OCRs measured in other cell culture systems. Finally, OCR data collected from the O-MCP enabled label-free detection of drug-induced metabolic shifts following exposure of hRPTECs to three mitochondrial toxicants tested in parallel on the same microtiter plate. Our system for label-free monitoring of OCR and metabolic shifts in an array of membrane bilayer devices contained within a microtiter plate format will serve as a valuable tool for data collection during pharmacological studies and disease research.

## Results

### O-MCP design and oxygen measurement setup

**Fig. 1:**
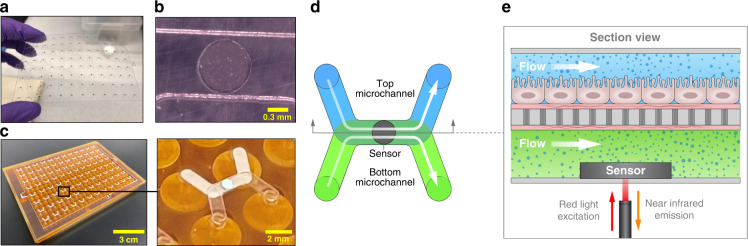
O-MCP fabrication and oxygen measurement setup. **a** Oxygen sensor units bonded to each of the 96-bottom microchannels of the O-MCP microfluidic layer. **b** A sensor unit bonded centrally to the floor of a single bottom microchannel. **c** Bottom view of the O-MCP with 96 devices and a zoomed-in image of a single device showing four inlet and outlet wells connected to the top and bottom microchannels and a sensor unit in the bottom microchannel (inset). **d** Top view of a single O-MCP device illustrates top and bottom microchannels with programmable flow rates, shown as white arrows, and an integrated oxygen sensor (black spot). **e** Cross-section side view of an O-MCP device illustrates continuous flow and oxygen (blue dots) distribution in two microchannels with hRPTECs cultured on the top surface of the porous membrane. An optical fiber positioned below the bottom microchannel delivers red excitation to the sensor unit and collects near-infrared emission with an oxygen-dependent phase shift

The O-MCP was designed as a 96-device array configured to collect oxygen measurements during controlled fluid flow. Each O-MCP device was a membrane bilayer device consisting of two microchannels separated by a porous membrane. Fluid flow was controlled in each microchannel via the micropump array housed in the O-MCP lid. The oxygen sensor units, 0.75 mm in diameter and 50 μm-thick, were bonded to the center of each of the 96 bottom microchannels, as shown in Fig. 1a, resulting in a sensor unit for each device, as shown in Fig. 1b, within the O-MCP microfluidic layer. The microfluidic layer was attached to a custom bottomless 384-well plate to create the full O-MCP, as viewed from the bottom in Fig. 1c. The inset of Fig. 1c. shows a bottom view of a single O-MCP device containing four inlet and outlet wells connected to the top and bottom microchannels and a sensor unit in the bottom microchannel. A single device of the O-MCP, shown schematically in Fig. 1d, allowed flow, controlled by the micropump array, to be administered to both microchannels with independent fluid streams. Oxygen consumption from hRPTECs cultured on the membrane in the top microchannel led to changes in oxygen concentration, which were measured via the oxygen sensor unit, illustrated in Fig. 1e. An oxygen meter and optical fiber positioned below each sensor unit delivered excitation at 610–630 nm to the sensor unit and collected emission at 760–790 nm with an oxygen-dependent phase shift.

### Sequential measurement of oxygen depletion across an array of membrane bilayer devices

**Fig. 2:**
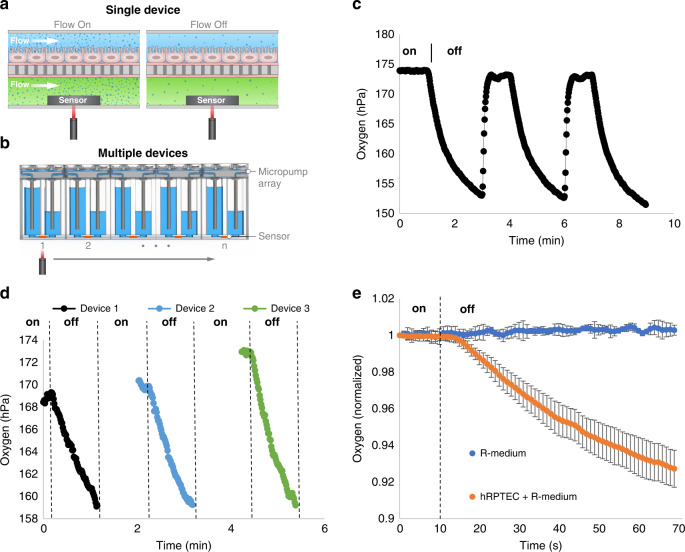
The O-MCP and corresponding measurement technique allowed single devices to be measured repeatedly or multiple devices to be measured sequentially. **a** Turning flow off within a single device resulted in a decrease in oxygen due to hRPTEC oxygen consumption. **b** Sequential alignment of an optical fiber beneath each device and modulation of flow on (on) and flow off (off) enabled oxygen depletion measurements for **c** a single device during repeat measurements or **d** multiple devices in an array. **e** Oxygen measurements in devices containing hRPTEC and R-medium compared to control devices containing R-medium without cells. Data are presented as mean ± standard deviation of *n* = 15 devices containing hRPTECs and three devices containing R-medium without cells. Data from each device was normalized to its initial time point

We developed a method to measure oxygen depletion in the O-MCP array that contained hRPTECs adhered to the porous membrane. We produced temporary flow-on and flow-off conditions in each of the 96 O-MCP devices simultaneously by programming the micropump array to turn on pumping at 70 μL/min or stop pumping. Turning flow off in each microchannel resulted in a decrease in oxygen near the sensor due to cell oxygen consumption, the low oxygen permeability of the COP microchannels, and the high oxygen permeability of the porous membrane, as shown schematically in Fig. 2a. Sequential alignment of an optical fiber beneath each device and repeatedly turning flow on and off enabled measurement of oxygen depletion for multiple O-MCP devices, shown schematically in Fig. 2b. Real-time oxygen measurements were taken in a single device while the micropump array was turned on and off three consecutive times; we observed a decrease in oxygen while flow was off and a return to the baseline oxygen level when flow was turned back on. A representative plot of the changes in oxygen for one of the devices is shown in Fig. 2c. The oxygen concentration remained steady at 174 hPa, while pumps maintained 70 μL/min of media flow but decreased to 153 hPa within 3 minutes once the flow was turned off. The oxygen concentration returned to 174 hPa within 1 minute of the micropump array being turned back on. Repeated modulation of flow on for 1 minute and flow off for 1 minute coupled with indexing the optical fiber to different sensor units enabled measurement of oxygen depletion across 15 devices containing hRPTEC and hRPTEC culture medium (R-medium). Figure 2d. shows oxygen depletion for three representative devices measured consecutively during sequential measurements of 15 devices containing hRPTEC and R-medium. Initial oxygen levels prior to turning flow off were below the saturation level of 200 hPa, which was expected due to oxygen consumption from cells in the top microchannel outcompeting the oxygen supply via the pumps. Initial oxygen levels also varied slightly within the range of 165–200 hPa, which was attributed to variability in the biology within each device, such as the number of cells, location of cells within each device, and cell metabolic activity. A comparison between oxygen depletion in devices containing hRPTECs and R-medium and control devices containing R-medium without cells is shown in Fig. 2e. Oxygen remained steady at initial levels within the control devices following turning off the micropump array. In devices containing hRPTECs and R-medium, oxygen remained steady at the initial levels for 4 s after turning off the micropump array, which was attributed to residual flow in each microchannel and a diffusion distance between the cell layer and sensor.

### Estimation of cell OCRs in the O-MCP

**Fig. 3:**
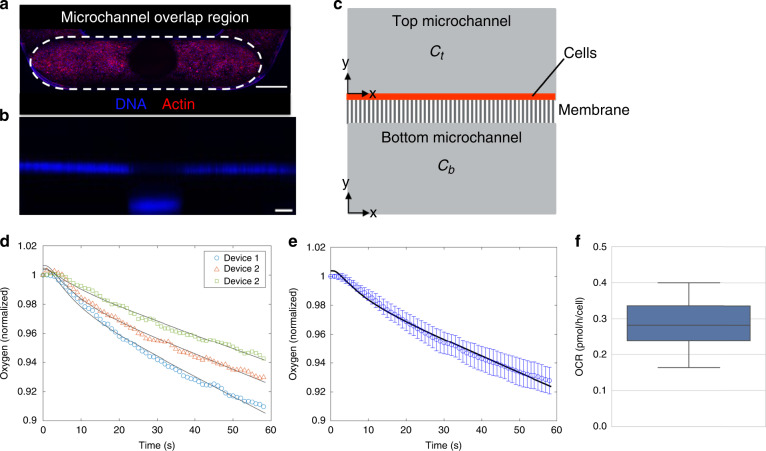
Finite element analysis-based modeling resulted in estimation of cell OCR in each O-MCP device. **a** View of the microchannel overlap region with hRPTEC stained for DNA (blue) and actin (red) (scale bar: 500 μm); cells showed uniform density. **b** Cross-sectional side view along the length of the overlap region showed cells stained for DNA along the membrane and autofluorescence from the sensor unit located on the floor of the bottom microchannel (scale bar: 200 μm). **c** Simplified 2D geometry for modeling oxygen transfer in the microchannel overlap region. **d** Representative experimental oxygen depletion (symbols) and numerically fitted (black line) curves were in close agreement. **e** Averaged experimental oxygen depletion data (blue circles) and numerically fitted (black line) curve used to estimate the hRPTEC OCR. Data are presented as mean ± standard deviation of 15 devices containing hRPTECs and R-medium. Data were normalized to the initial experimental time point for visualization purposes. **f** Estimated hRPTEC OCR as a result of the curve fitting procedure

A mass transfer model describing oxygen depletion in each O-MCP device allowed estimation of OCRs for cells cultured on the membrane. In each O-MCP device, hRPTECs formed a uniform layer on the central porous membrane in the top microchannel with high viability (Fig. S1). Figure 3a. shows hRPTECs stained for DNA and actin located on the top side of the membrane in the microchannel overlap region containing the centrally located light-absorbing sensor unit, shown as a dark spot. A cross-sectional view of the microchannel overlap region in Fig. 3b. shows hRPTECs stained for DNA along the membrane and auto fluorescence from the sensor unit located on the floor of the bottom microchannel. A 2D mass transfer model following Fick’s law was proposed to simulate oxygen changes occurring in the microchannel overlap region as a result of cell oxygen consumption on the top surface of the membrane. The microchannel overlap region was discretized into two components: a top microchannel containing cells at the bottom surface and a bottom microchannel with no cells, as shown in Fig. 3c. Oxygen transfer between the COP microchannel walls and media was neglected due to the low oxygen diffusivity in COP^36^. Because the experimental curves were nonlinear (Fig. 2c), we proposed a model that allowed for an initial oxygen gradient between the top and bottom microchannels. Due to localization of cells to the top microchannel (including inlets and wells), oxygen concentrations were expected to be lower in the top microchannel than in the bottom microchannel prior to turning flow off. By assuming symmetry along the microchannel length, all governing and boundary equations could be simplified to 1D differential equations. The governing equations following Fick’s law in the top and bottom microchannels are given by

1 $$\frac{{\partial C(y,t)_i}}{{\partial t}} = D \ast \frac{{\partial ^2C(y,t)_i}}{{\partial y^2}} \quad i = T,B$$

where the oxygen concentrations in the top and bottom microchannels are *C*_*T*_ and *C*_*B*_, respectively, *D* is the oxygen diffusion coefficient in the cell culture medium, *y* is the position along the height of each microchannel, and *t* is time. In the top microchannel, oxygen is consumed by cells and transferred between the top and bottom microchannels across a membrane. Therefore, the boundary conditions for the top microchannel are summarized by

2 $$D \ast \frac{{\partial C(y,t)_T}}{{\partial y}}|_{y = h_T} = 0$$

3 $$\begin{array}{ll}D \ast \frac{{\partial C\left( {y,t} \right)_T}}{{\partial y}}|_{y = 0} = \frac{{D_M}}{{h_M}} \ast \left[ {C_T\left( {0,t} \right) - C_B\left( {h_B,t} \right)} \right] \\+ \frac{{V_m \ast \varphi \ast C_T\left( {0,t} \right)}}{{K_m + C_T\left( {0,t} \right)}}\end{array}$$

where *h*_*T*_ and *h*_*B*_ are the top and bottom microchannel heights, respectively. The membrane was modeled as a thin diffusion barrier^37^, where *D*_*M*_ is an effective membrane diffusion coefficient and *h*_*M*_ is the membrane thickness. Renal cell oxygen consumption was modeled based on Michaelis‒Menten kinetics^38^, where *V*_*m*_ is the maximum cell OCR, *φ* is the cell surface density on the membrane, and *K*_*m*_ is the Michaelis constant.

In the bottom microchannel, there exists a flux at the top surface due to oxygen transfer between the top and bottom microchannels across a membrane. Therefore, the boundary conditions for the bottom microchannel are summarized in the following equations:

4 $$D \ast \frac{{\partial C(y,t)_B}}{{\partial y}}|_{y = h_B} = \frac{{D_M}}{{h_M}} \ast \left[ {C_T\left( {0,t} \right) - C_B\left( {h_B,t} \right)} \right]$$

5 $$D \ast \frac{{\partial C(y,t)_B}}{{\partial y}}|_{y = 0} = 0$$

The above time-dependent equations were solved numerically using the forward Euler method^39^ in a homemade MATLAB modeling environment with the input parameters provided in Table S1. The maximum cell OCR (*V*_*m*_) and initial oxygen concentrations, *C*_*T*_ (*y*,0) and *C*_*T*_ (*y*,0), were extracted by numerically fitting the mass transfer model to the experimental oxygen depletion curves. The curve fitting procedure was carried out via minimization of the residual sum of squares between the predicted and experimental curves using the objective function given by

6 $$J = \mathop {\sum }\limits_{i = 1}^N \left[ {C_{exp,i} - C_{pred,i}} \right]^2$$

where *N* is the number of time points, *C*_*exp,i*_ represents the experimental concentration at the *i*th time point, and *C*_*pred,i*_ is the predicted concentration at the floor of the bottom microchannel at the *i*th time point. Experimental oxygen measurements, in units of partial pressure, were converted to concentration with Henry’s law and assuming an oxygen solubility in cell culture medium of 215 μM^37,40^ to enable comparison with predicted curves. The curve fitting procedure resulted in simulated oxygen depletion curves that fit the experimental curves closely, as shown in data from three representative devices in Fig. 3d. and data averaged over 15 devices in Fig. 3e. The curve fitting procedure resulted in an estimated OCR for hRPTECs of 0.29 ± 0.07 pmol/h/cell, as shown in Fig. 3f, for quantification of cell metabolic activity and comparison with OCRs from other systems.

**Fig. 4:**
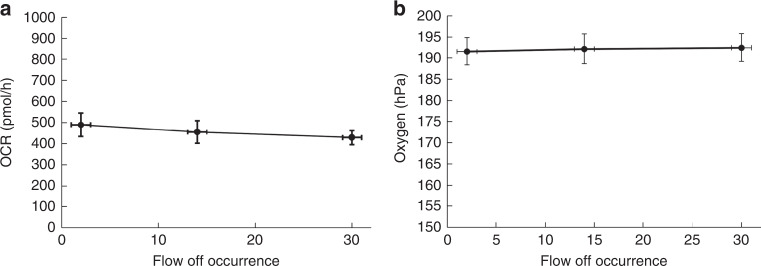
Repeated modulation of flow on and off did not significantly alter the OCR and oxygen measurements. **a** OCR and **b** oxygen measurements under flow during repeated pump stops. Data are presented as mean ± standard deviation of *n* = 3 devices containing hRPTECs

Because flow was repeatedly turned on and off during sequential device measurements, we evaluated the consistency of single device OCR readouts during repeated modulation of flow on and off. We monitored the OCR for three devices containing hRPTEC and R-medium during 31 repetitions of turning flow on at 70 μl/min for 1 minute and off for 1 minute. The OCR remained consistent across the 31 flow-off occurrences, as shown in Fig. 4a, which indicated that turning the flow on (at 70 μl/min) and off had a negligible effect on a single device’s OCR readouts. Additionally, oxygen readings measured during flow-on conditions, shown in Fig. 4b, remained consistent over the course of 31 flow-off occurrences. Therefore, repeated modulation between flow-on and flow-off conditions, necessary to acquire readings across an array of O-MCP devices using a single optical fiber, had a negligible effect on individual device OCR readouts, ensuring negligible error due to the technique.

### Detection of drug-induced shifts in mitochondrial function

**Fig. 5:**
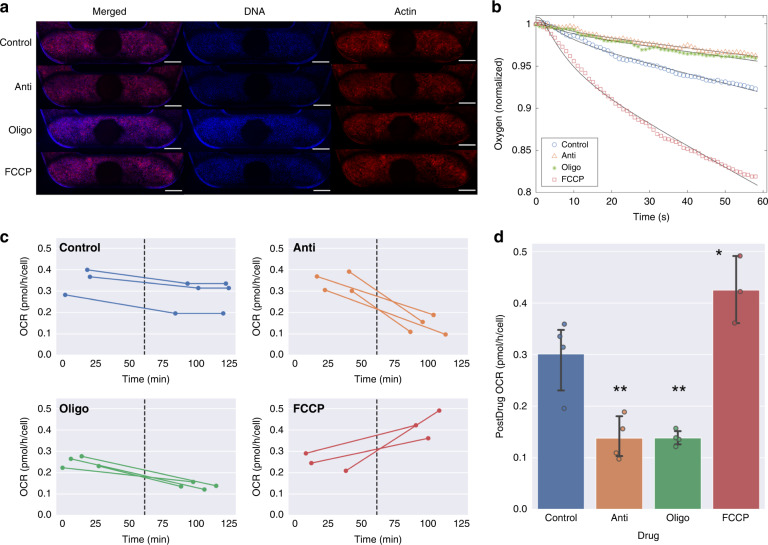
Detection of drug-induced shifts in mitochondrial function. **a** Representative images of hRPTECs stained for nuclei (blue) and actin (red) showed no significant changes in cell density following a 1.5-hour exposure to either 1.5 μM Antimycin A (Anti), 1.5 μM Oligomycin (Oligo), or 2 μM FCCP (scale bar: 500 μm). **b** Representative oxygen depletion curves (symbols) with simulated curve fits (black line) for single devices in the control, Anti, Oligo and FCCP groups. **c** Single-device OCR measurements acquired pre- and postdrug exposure at *t* = 61.4 minutes (dotted line). **d** OCR following drug exposure for each condition. Data are from 3–4 devices per condition. Error bars represent standard deviation, *p < 0.05, **p < 0.01 with respect to control (single-factor ANOVA with Tukey’s Post Hoc test)

hRPTECs cultured under flow at 70 μl/min for 6 days responded to 1.5 μM antimycin A (Anti), 1.5 μM oligomycin (Oligo) and 2 μM trifluoromethoxy carbonyl cyanide phenylhydraxone (FCCP), which are drugs that directly interfere with mitochondrial function. Anti and Oligo, which are mitochondrial oxidative phosphorylation inhibitors, reduce cell oxygen consumption, and FCCP, a mitochondrial oxidative phosphorylation uncoupler, increases oxygen consumption^41^. Representative images of hRPTECs stained for nuclei and actin following a 1.5-hour exposure to Anti, Oligo, and FCCP (Fig. 5a) showed no significant differences in cell density following drug exposure (see Fig. S2). Oxygen depletion curves were acquired in devices pre- and postdrug treatment for estimation of the cell OCR via curve fitting. We found that all oxygen depletion curves and corresponding curve fits used to estimate cell OCR were in close agreement (Fig. S3). Representative oxygen depletion curves measured in devices treated with Anti, Oligo, FCCP, and no drug (control) are shown in Fig. 5b. and displayed differences in oxygen consumption as a result of drug activity. Figure 5c. shows the OCR measurements of individual devices before and after drugs were administered at *t* = 61.4 minutes, indicated by the dashed line in the plots, for each condition. Control devices with no drugs administered, shown in the top left graph of Fig. 5c, were measured at three time points to measure any potential changes in the OCR due to nondrug-related changes in biological activity or due to experimental handling of the system. Control readings acquired before, during, and after measurements of devices containing drugs indicated a slight decrease in the OCR. The postdrug OCR relative to the predrug OCR was computed for each device (using the first and second time points for control measurements), as shown in Fig. 5c. The postdrug OCR relative to the predrug OCR was 80% for the control, 40% for Anti, 56% for Oligo, and 176% for FCCP conditions, as reported in Table 1. Postdrug OCRs, shown in Table 1 and Fig. 5d, indicated a significant reduction in oxygen consumption due to both inhibitors (*p* < 0.01) and a significant increase in oxygen consumption due to FCCP, the uncoupler agent (*p* < 0.05).

**Table 1:** Summary of OCR measurements for different drug exposures (*n* = 3–4 devices)

Drug	Postdrug OCR (pmol/h/cell)	Postdrug OCR relative to predrug OCR (%)
Control	0.30 ± 0.07	79.6 ± 9.0
Antimycin A	0.14 ± 0.04	39.8 ± 8.3
Oligomycin	0.14 ± 0.01	56.2 ± 10.8
FCCP	0.42 ± 0.07	175.9 ± 51.5

## Discussion

The O-MCP had integrated sensor units in each of the 96 OOC devices, which enabled the collection of oxygen concentration data in each device one at a time via the sequential positioning of a single optical fiber below each sensor prior to measurements. The state of the art commercial Agilent Seahorse XFeAnalyzer uses 96 probes inserted into all wells of a standard 96-well plate for measurement of oxygen consumption^42^ but does not support perfusion and is not compatible with OOC architectures. In contrast, the O-MCP is a sensor-integrated OOC system that allows oxygen measurements during controlled perfusion in membrane bilayer devices used to study complex tissue models^3,43,44^. In addition to oxygen sensing, the design and fabrication of the O-MCP is easily adaptable for other sensing applications. Optical access to the bottom microchannel and a simple sensor bonding process allow for other optically based sensor units to be integrated that measure other critical cell culture parameters, including pH^45^ and glucose^46^. Additionally, for the measurement of cell culture parameters across the entire channel length while maintaining optical access for microscopic imaging, transparent liquid sensors could be deposited along the entire bottom microchannel via spray coating, similar to the procedure reported in Ehgarter et al.^23^. Finally, the O-MCP sensor integration has minimal hardware requirements because it easily integrates with a standard microscope, utilizing the objective turret and programmable stage for alignment of a single optical fiber beneath each sensor prior to measurements in each device. Additionally, the oxygen sensors integrate well with impedance-based sensors for the measurement of transepithelial-endothelial electrical resistance, as shown in previous work^29^, because there is no interference between the optical and electrical signals. For integration of multiple optical-based sensors that measure different biological parameters, such as pH or glucose, the issue of signal specificity should be further explored due to the coexistence of multiple optical signals in the same environment.

Previous groups have integrated optical-based oxygen sensors into single OOC devices and measured transient oxygen depletion by modulating the flow on and off^45^ and during steady-state flow-on conditions^20^; however, data were limited to only one or a few individual devices in parallel. The O-MCP demonstrated the capability to collect OCR data from an array of OOC devices contained in an industry-standard microtiter plate format via sequential positioning of a single optical fiber beneath each device, repeated modulation of flow on and off, and acquisition of transient oxygen depletion measurements. Additionally, in contrast to previous works, the O-MCP enabled oxygen measurements for cells cultured on a membrane between two microchannels, which is a setup well suited for studying tissue barriers and provides a mechanism to separate cells from the sensor unit. Separation of cells from the sensor unit was critical to ensure that cells formed a confluent tissue layer without obstruction from the sensor. To our knowledge, existing OCR measurement systems^24,27,28^ have not utilized a porous membrane to separate cells from the sensor. We also found that turning flow off for a short duration of 1 minute was sufficient to detect oxygen depletion at the sensor location due to cell oxygen consumption along the membrane. The capability to detect significant depletion in oxygen within a short measurement period was particularly useful, as it enabled sequential measurements across multiple devices situated in parallel with moderate speed (15 devices/30 minutes) using only a single optical fiber and a programmable microscope stage. Integration of an array of optical fibers with the high-throughput OOC platform would increase the data collection speed and be compatible with the current procedure.

Measurement of the cell OCR in OOC systems enables comparison with metabolic rates from different cell culture systems, improved sensitivity to biological changes and correction for nonbiological contributions to the measurements. While several studies have measured the cell OCR in single microchannels^27,28,45^, it remains a challenge to do so in multilayer devices and 3D cultures because the cells and integrated sensors are generally not confined to a single fluidic chamber with well-defined geometry and diffusion characteristics. Therefore, finite element analysis-based modeling has proven useful for the simulation of oxygen distributions^37^ and estimation of the cell OCR^20^ in OOC systems. Bilayer membrane devices in particular have emerged as a useful cell culture format^8,31^, yet current systems have not demonstrated OCR measurements. For the first time, to our knowledge, we employed a finite element analysis-based model and curve fitting approach to estimate OCR for cells localized to a porous membrane suspended between two microchannels. As a proof of concept, the OCR was measured for only one cell type cultured on the central membrane. Future work should investigate the measurement of OCR for tissue models in which two or more cell types are cultured on the membrane.

The measured OCR of 0.29 pmol/h/cell for hRPTECs in the O-MCP was in the range of human cell OCR values reported in other in vitro studies^20,47^ and ~six times lower than a previously reported measurement of hRPTEC OCR^38^. The difference between our measured OCR for hRPTECs and the previously reported value is likely due to differences in culture conditions, particularly glucose concentrations, between the two culture systems. In support of this claim, Beeson et al.^48^ previously demonstrated that medium conditions, particularly glucose concentrations, can heavily influence primary renal epithelial cell OCR. The 176% FCCP-induced increase in the hRPTEC OCR was similar to a previously reported 189% increase in hRPTEC OCR following treatment with CCCP, a mitochondrial uncoupler compound similar to FCCP^38^. Similar to previous in vitro studies^48,49^, Anti and Oligo decreased cell OCR; however, the magnitudes of the shifts differed relative to the literature values, which we attributed to differences in biological conditions, including cell types and culture mediums.

For the first time, to our knowledge, we measured drug-induced shifts in cell OCR for human renal epithelial cells cultured on a porous membrane between two microchannels and exposed to unidirectional perfusion for several days, mimicking the flow conditions in the kidney. Existing oxygen sensor-integrated OOC systems used for the measurement of drug-induced OCR shifts have been limited to one device per drug condition^20,27,45^. Our work demonstrates an improvement in throughput capability for drug investigations in OOC systems by measuring the OCR responses for three different drug treatments, in addition to a control group, with a minimum of three devices per condition in a single microtiter plate format. We found that the measurement of control devices before, during, and after drug exposure was particularly important for assessing whether changes in OCR were a result of drug action or common error sources, such as variability in cell number, cellular changes over time or due to disturbance, or other environmental changes, such as temperature or pH. It was only necessary to use 16% of the devices arrayed within the O-MCP for proof of concept work here. However, the remaining capacity of the O-MCP presents an immediate opportunity to include additional doses, drugs, biological conditions, or other parameters that would expand the scope of the experiment. Additionally, in future work, an increase in throughput could be realized by automating the sequential measurement procedure described in this work and allowing for a duration of 1.6–3.2 hours per plate (1–2 minutes/device). Alternatively, incorporating an array of optical fibers that collect data from each sensor unit simultaneously would increase the data collection speed and allow for measurement in all 96 devices within 1--2 minutes. In future work, achieving full automation and fast data collection will also enable many samples to be collected in a single device under the same conditions, which will allow thorough evaluation of the reproducibility of biological data collected in OOC studies. In a recent study, large sample sizes collected under controlled conditions enabled evaluation of data reproducibility for a cell migration assay^50^.

In conclusion, we presented an oxygen sensor-integrated OOC platform (O-MCP) and an approach to measure OCR in an array of membrane bilayer devices contained within a microtiter plate format. The integrated sensing platform and measurement approach enabled monitoring of cell metabolic activity for perfused tissues in an industry-standard format that is compatible with life science infrastructure such as high-content imaging. Data collection from the O-MCP did not significantly alter the OCR measurements, as demonstrated by the negligible effect of repeatedly modulating flow on and off on individual device readouts. Finite element analysis-based modeling resulted in the estimation of single-cell OCR data, allowing comparison with cell metabolic rates measured in other cell culture systems. Finally, we demonstrated the sensitivity of the OCR measurements for label-free detection of metabolic shifts in hRPTECs following exposure to three different drugs known to interfere with mitochondrial function. The O-MCP system is capable of collecting label-free metabolic data in an array of membrane bilayer devices contained within a microtiter plate format, which will serve as a powerful tool for studying drugs and disease in flow-based tissue models.

## Materials and methods

### O-MCP and micropump array

Optical oxygen-sensitive sensor units (0.75 mm in diameter and 50 micron thick) were cut with a biopsy punch from a 25 cm^2^ sheet of photosensitive oxygen sensor foil (item No. OXFOIL-TN, Pyroscience, Germany). Integration of sensor units and fabrication of the O-MCP assembly has been previously described in detail^29^. Briefly, 188 μm-thick cyclic olefin polymer (COP) (ZF14-188: Zeon Corp., Tokyo, Japan) with 96 laser-cut microchannels was laminated to 188 μm-thick COP in a heated hydraulic press (Carver Inc., Wabash, IN, USA) at 120 °C and 1 MPa for 30 minutes to form a bottom microchannel layer. Sensor units were positioned in the center floor of each bottom microchannel with forceps and bonded using silicone adhesive (Sylgard 184, Corning). Lamination of 188 μm-thick COP to a 188 μm-thick COP layer with 384 holes positioned at the inlet and outlets of each microchannel formed the top microchannel layer. An 11 μm-thick polycarbonate track-etched membrane with 1 μm pores (Sterlitech, WA, USA) was patterned using a UV laser system (Protolaser U4: LPKF Laser and Electronics, Garbsen, Germany) with 384 holes positioned at the inlets and outlets of each microchannel. The membrane was laminated between the bottom and top microchannel layers to form the microfluidic stack. The microfluidic stack was bonded with a pressure-sensitive adhesive to the bottom of a custom bottomless 384-well plate to form the O-MCP. The fabrication of a 192 micropump array that generated controlled flow rates in the top and bottom microchannels of each O-MCP device was done as previously described^29^. Briefly, the micropump array consisted of a pneumatic manifold layer, a membrane, and a fluidic circuit consisting of an intake valve, a pump chamber, and an expulsion valve. The micropump array sat on the O-MCP and interfaced with custom software to enable programmable flow rates ranging from 1–70 μL/min in each microchannel.

### Cell culture

hRPTEC culture medium (R-medium) was prepared by supplementing 50:50 DMEM/F12 (Gibco, USA) with 0.5% fetal bovine serum (Life Technologies, USA), 10 ng/mL recombinant human epidermal growth factor (Thermo Fisher, USA), 5 µg/mL insulin (Sigma‒Aldrich, USA), 0.5 µg/mL hydrocortisone (Sigma‒Aldrich, USA), 0.5 µg/mL (±)-epinephrine (Cayman Chemical, USA), 6.5 ng/mL 3,3’,5-triiodo-L-thyronine sodium salt (Sigma‒Aldrich, USA), 10 µg/mL transferrin, human (Sigma‒Aldrich, USA), and 1% antibiotic-antimycotic (Life Technologies, Canada). Primary hRPTECs (ScienCell, Lot: 5340) and R-medium were cryopreserved at −190 °C in cryogenic vials at a density of 1 million cells/mL. Two days prior to seeding cells in the O-MCP microchannels, a 1 mL cryopreserved solution of hRPTECs was thawed and transferred to a T-150 flask (Thermo Fisher, USA) with 30 mL of R-medium. The flask was kept in an incubator at 37 °C, 50% humidity, and 5% CO_2_, and the medium was exchanged after 24 hours. On the day of seeding, hRPTECs reached approximately 90% confluency and were then rinsed with phosphate-buffered saline (PBS) (Thermo Fisher, USA), detached with 5 mL of 0.25% trypsin-EDTA (Life Technologies, USA), diluted in 10 mL of R-medium, and transferred to a 10 mL conical tube. hRPTECs were counted, and then the 10 mL cell solution was centrifuged at 250 g for 5 minutes to form the supernatant. The R-medium above the supernatant was aspirated, and the cells were resuspended in R-medium to form a one million cells/mL solution.

### Cell Seeding

The O-MCP microchannels were sterilized with ethylene oxide gas and then degassed for 2 days. The microchannels were exposed to oxygen plasma treatment for 1 minute to increase the hydrophilicity of the microchannel surfaces. Microchannels were filled with ethanol, remained filled for 20 minutes, and then rinsed three times with PBS. Microchannels were then filled with a solution of 60 μg/mL collagen from human placenta (Sigma Aldrich, USA) in PBS, and the culture plate was rocked for 1 hour at room temperature. Following three PBS rinses, 15 μL of R-medium was delivered to all microchannel inlet and outlet wells. Medium was aspirated from the top inlet well prior to cell seeding. Thirty microliters of a one million/mL solution of hRPTEC in R-medium was pipetted into the inlet well of each top microchannel. One hour following seeding, R-medium was replaced with 60 μl of fresh medium in all four inlet and outlet wells of each microchannel. Micropump array-driven flow started in all microchannels at 10 μl/min. Two days after seeding, the flow rate increased to 70 μl/min for the remainder of the experiment. R-medium changes occurred every 2 days and 1 hour prior to all oxygen measurements.

### Oxygen measurements

A FireStingO2 optical oxygen meter (Pyroscience, Germany) with an optical fiber was used to measure oxygen in each O-MCP device. During oxygen measurements, the O-MCP was placed on the stage of a confocal microscope with a cell culture incubation chamber (LSM700, Zeiss), and the optical fiber was secured parfocally onto the objective turret using a custom fixture (Fig. S4). Because the distance between the optical fiber and sensor unit influences the oxygen measurements, it was critical that the distance remained consistent for different devices. Therefore, the microscope’s 10x objective and programmable stage was used to visually locate each sensor unit and position the optical fiber beneath each sensor unit at a consistent distance for different devices. The programmed stage positions were used to align the optical fiber with each sensor unit during measurements. A typical 2-point calibration was performed in FireStingO2 software using microchannels filled with water for the “100% saturated” solution and microchannels filled with 30 g/L sodium sulfite in deionized water for the “0% saturated” solution. Single-device oxygen depletion measurements were acquired at a continuous 1 Hz sampling rate as flow at 70 µL/min was turned on within the top and bottom microchannels for 10 seconds and then turned off for 1 minute.

### Drug treatment

The drugs antimycin A (Anti), oligomycin (Oligo), and carbonyl cyanide 4-(trifluoromethoxy)phenylhydrazone (FCCP) in powder form were purchased from Abcam (Cambridge, UK), and dimethyl sulfoxide (DMSO) was purchased from Sigma Aldrich (St. Louis, MO). All drugs were diluted in DMSO to produce 8 mM Anti, 3 mM Oligo, and 8 mM FCCP stock solutions. The drug-DMSO stock solutions were further diluted in R-medium to produce drug-R-medium solutions of 18 μM Anti, 18 μM Oligo, and 24 μM FCCP. Prior to the acquisition of oxygen data following drug treatments, the micropump array was lifted off of the O-MCP, and 10 μL of each drug-R-medium solution was pipetted into the inlet and outlet wells of the top and bottom microchannels to produce final solutions of 1.5 μM Anti (0.02% DMSO), 1.5 μM Oligo (0.05% DMSO), and 2 μM FCCP (0.025% DMSO) in R-medium within each microchannel. Following drug exposure, the flow rate was set to 70 μL/min in both microchannels for 23 minutes prior to OCR measurements.

### Fluorescent staining and microscopic image acquisition

Live cells were stained using a 1 mg/ml stock solution of calcein AM (Abcam, USA) diluted to 1:1000 in PBS. For fluorescent staining, cells were rinsed with cold PBS three times. Cells were then fixed with 4% paraformaldehyde (Thermo Fisher) in PBS for 10 minutes followed by three rinses with PBS. Cells were permeabilized with 0.1% Triton-X (Thermo Fisher, USA) with PBS for 5–10 minutes at room temperature followed by three PBS rinses. Cell nuclei were labeled with a 1 mg/mL solution of Hoechst 33342 (Thermo Fisher, USA) diluted to 1:500 in PBS. Actin was labeled with phalloidin 647 (Thermo Fisher, USA) diluted to 1:1000 in PBS. Images were acquired with a scanning confocal microscope (LSM700, Zeiss) using ×10 air, ×20 air, and ×40 water immersion objectives and 405 nm, 488, and 637 nm lasers.

### Cell count

Images of nuclei for 4328 × 328 μm^2^ regions of interest were captured along the membrane of each top microchannel. Manual nuclei counts of the 328 × 328 μm^2^ images were performed using a Fiji plug-in titled Cell Counter^51^ and averaged for each microchannel. Each microchannel’s average nuclei count was divided by the surface area of the 328 × 328 μm^2^ region of interest to compute cell density. The total number of cells along the membrane in the microchannel overlap region was computed by multiplying the cell density by the surface area of the top surface of the membrane.

### Statistical analysis

Single-factor analysis of variance and Tukey’s post hoc analysis were used to calculate significant differences between different groups. All *n* values represent the number of devices measured per condition. All error bars represent ± standard deviation.

## Supplementary information

Supplemental Material

## Data Availability

Data that support the findings of this study are available from the corresponding authors upon reasonable request.
