# Supplementary material for: Measurement of oxygen consumption rates of human renal proximal tubule cells in an array of organ-on-chip devices to monitor drug-induced metabolic shifts
Source: Microsyst Nanoeng. 2022 Sep 29;8:109. doi: 10.1038/s41378-022-00442-7 (PMC9519964; doi:10.1038/s41378-022-00442-7)
Supplement: Supplementary file 1 — Supplemental Material [file 41378_2022_442_MOESM1_ESM.docx]

**Supplementary Information**

**Figure S2**:

Cell nuclei count on the membrane in the top microchannel following 1.5 hour treatment with no drugs (Control), 1.5 μM Antimycin A, 2 μM FCCP, and 1.5 μM Oligomycin for 1.5 hours. There was no significant difference in nuclei counts between the different drug groups (p=0.08, single factor ANOVA). Data are mean ± standard deviation of 3-4 devices.

**Table S1: Input parameters for modelling oxygen transfer in the O-MCP**

| **Parameter** | **Value** | **Description** |
| --- | --- | --- |
| $D$ | 2.5x10^-5^ cm^2^/s | Diffusion coefficient for oxygen in cell culture medium^1^ |
| $h_{T}$ | 215 μm | Top microchannel height |
| $h_{B}$ | 215 μm | Bottom microchannel height |
| $P_{M}$ | 16 % | Membrane porosity |
| $D_{M}$ | 4.0x10^-6^ cm^2^/s | Effective membrane diffusion coefficient ($\frac{P_{m}}{100}* D$) |
| $h_{M}$ | 11 μm | Membrane thickness |
| $K_{m}$ | 60 μM | Michaelis constant for hRPTECs^2^ |

**Figure S1:**

a) hRPTECs, stained with calcein, formed a confluent and viable monolayer on the central porous membrane in the top microchannel (scale bar: 500 μm). b) Representative zoomed-in image of cell nuclei showed uniform cell density (scale bar: 25μm).

**Figure S3:**


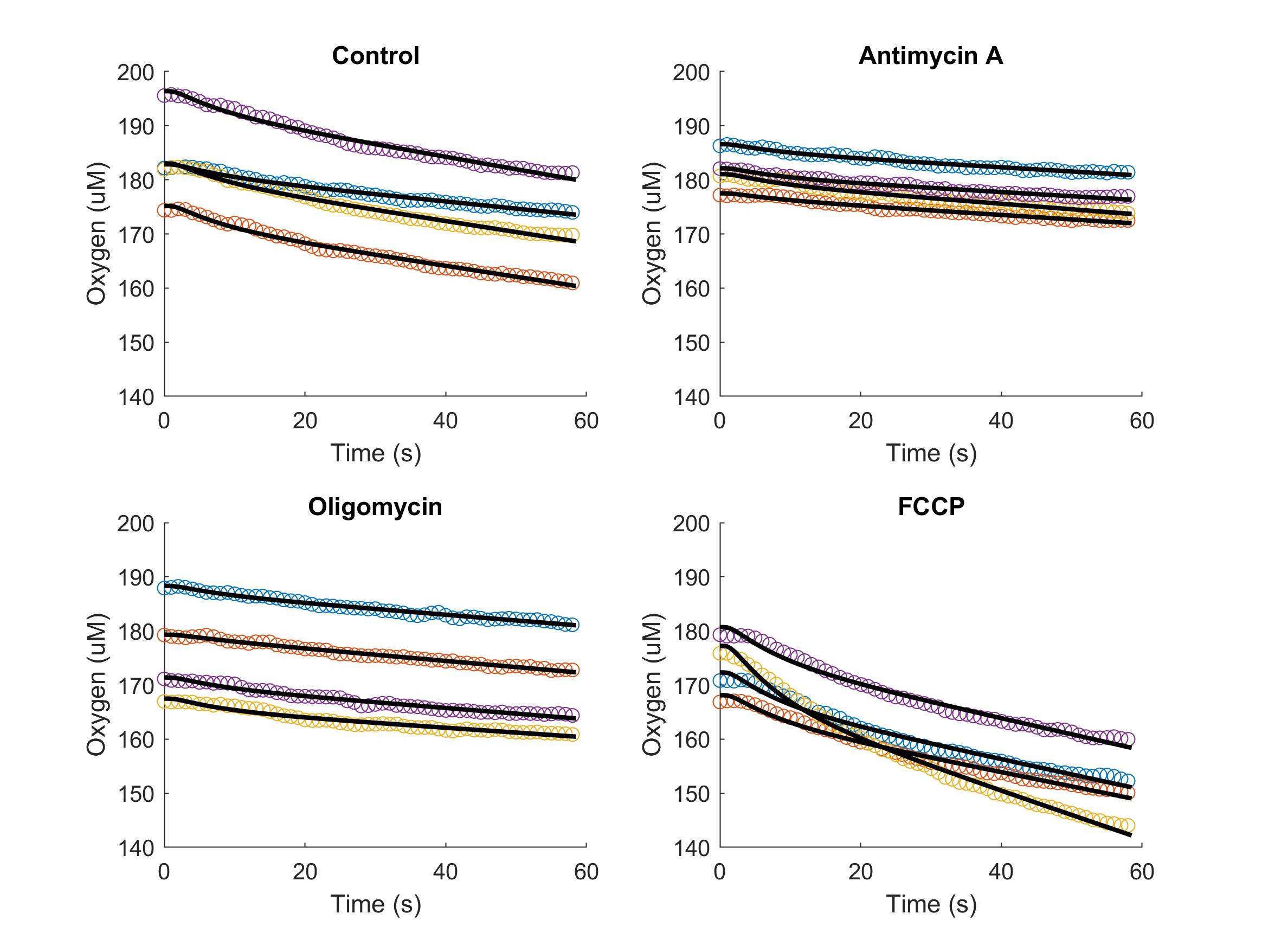


Experimental oxygen depletion curves (circles) and simulated curve fits (black lines) used for estimation of OCR following drug treatments. Colors correspond to different devices tested within each drug group.

**Figure S4: Experimental setup for oxygen measurements.**


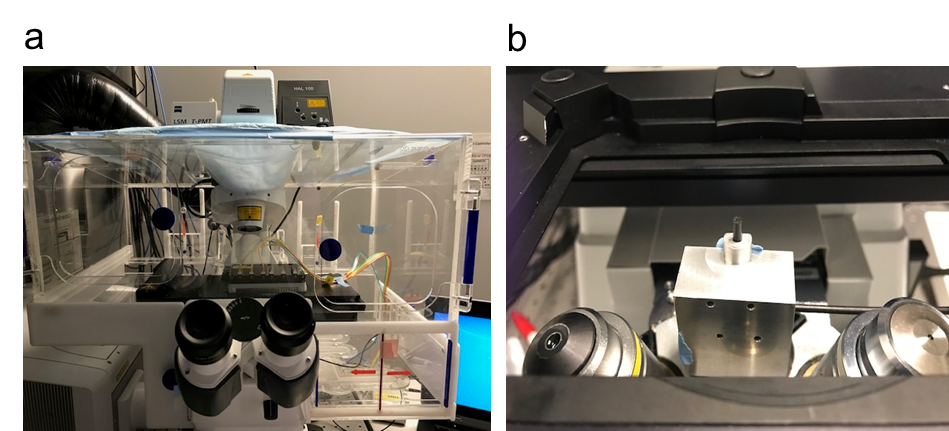


a) Confocal microscope equipped with a cell culture incubation chamber and a programmable stage. b) A custom fixture secured to an objective port positioned the fiber optic beneath each sensor during oxygen measurements.

**Figure S5: High and low calibration measurements in the O-MCP**


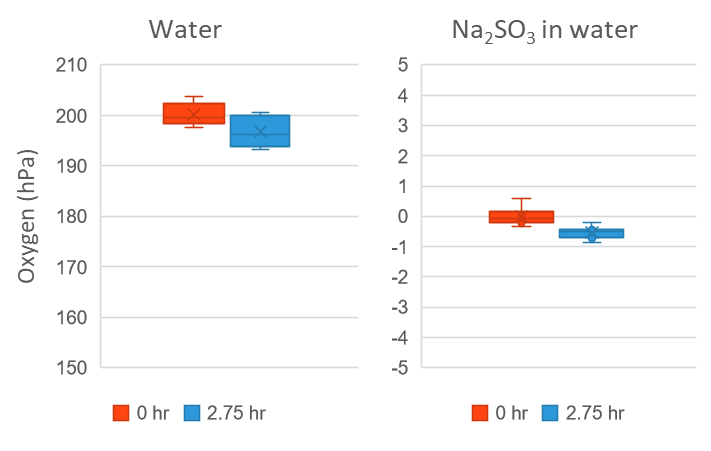


Oxygen measurements in calibration control devices filled with the a) 100% air saturated solution (water) and b) 0% air saturated solution (30 g/L sodium sulfite (Na_2_SO_3_) in water) at the time of calibration (t=0 hr.) and following 2.75 hours (t=2.75 hr.). A slight decrease of 3.4 and 0.54 hPa for the high and low solutions, respectively, was observed over the course of 2.75 hours. We found this error to be acceptable given that it was a relatively small change over a long time duration and that our technique measures oxygen changes on the timescale of seconds for computing consumption rates.

References

1. M. Zahorodny-Burke, B. Nearingburg and A. L. Elias, Chemical engineering science, 2011, 66, 6244–6253.
2. D. Luttropp, M. Schade, P. C. Baer and J. Bereiter-Hahn, Biotechnology Progress, 2011, 27, 262–268.
